# Impact of Heated Versus Unheated Cooking Oil on Postprandial Vascular Function and Metabolism

**DOI:** 10.1002/lipd.70052

**Published:** 2026-04-05

**Authors:** Rosiered Brownson‐Smith, Jo Bruce, Esmat Abbas, Sarah Fissler, Wendy Hall, Sarah Berry

**Affiliations:** ^1^ Department of Nutritional Sciences, School of Life Course Sciences, Faculty of Life Sciences & Medicine King's College London London UK; ^2^ PURA Foods Ltd Erith UK

**Keywords:** arterial stiffness, cardiovascular risk, endothelial function, flow‐mediated dilation, heated oils, postprandial lipaemia

## Abstract

The impact of repeated heating of seed‐based culinary oils on cardiometabolic health has not been well established. Heating oils to high temperatures (> 150°C) causes lipid peroxidation, thus generating potentially harmful compounds that may impair vascular function. This randomized, single‐blind, crossover study investigated the acute effects of a high‐fat meal containing either repeatedly heated (at 180°C, over 10 days, with 5 potato fries per day) sunflower oil‐palm olein blend, compared with an unheated blend (containing 23.8% and 7.2% polar compounds respectively), on postprandial lipids and vascular function in 19 healthy male participants. Participants consumed each test meal on separate days, with measurements of flow‐mediated dilation (FMD; primary outcome), arterial stiffness, plasma triacylglycerol (TAG), non‐esterified fatty acid (NEFA), and glucose concentrations over 4 h. Postprandial vascular (FMD and arterial stiffness) responses as well as NEFA and glucose concentrations were comparable between meals. The postprandial TAG increase was lower after heated versus unheated oil (*p* < 0.001). These findings indicate that repeated heating of oil modifies postprandial lipaemia without acutely impairing vascular function. However, these short‐term results do not address the potential cumulative effects of chronic, long‐term consumption.

**Abbreviations**
BHT: Butylated Hydroxytoluene
BMI: body mass index
CAIx: central augmentation index
CVD(s): cardiovascular disease(s)
EDTA: ethylenediaminetetraacetic acid
FMD: flow‐mediated dilation
GTN: glyceryl trinitrate
HPLC: high‐performance liquid chromatography
NEFA: non‐esterified fatty acids
PAIx: peripheral augmentation index
PPL: postprandial lipaemia
SPSS: statistical package for the social sciences
TAG: triacylglycerol(s)

## 1 Introduction

Globally, a large proportion of dietary fat intake is derived from fats and oils which have undergone heating at high temperatures. Repeated heating of oils may impact health due to compounds formed during heating, which may affect lipid peroxidation, inflammation, or vascular function (Leong 2021; Venkata and Subramanyam 2016; Soundararajan et al. 2023). However, the health effects in humans of consuming oils that have been repeatedly heated, as commonly occurs during deep‐frying in commercial food preparation, are uncertain.

Exposing oils to deep frying temperatures repeatedly (typically ~180°C) causes significant chemical changes, resulting in the formation of secondary oxidation products (e.g., aldehydes, ketones, malondialdehyde) (Latha and Nasirullah 2014; Tyagi and Vasishtha 1996; Yılmaz et al. 2023; Nayak et al. 2016) and the formation of dimers, polymers, and total polar compounds, which accumulate with repeated or prolonged heating (Latha and Nasirullah 2014; Tyagi and Vasishtha 1996; Zhang et al. 2012; Takeoka et al. 1997). Repeated heating accelerates the formation of other hazardous substances, including polycyclic aromatic hydrocarbons and acrylamide, some of which are carcinogenic (Latha and Nasirullah 2014; Choe and Min 2007). Heating of oils can also lead to the degradation of minor components beneficial for health, including natural antioxidants (e.g., tocopherols, carotenoids) (Latha and Nasirullah 2014; Krishna and Swamy 2023).

It has been proposed that these chemical changes following repeated heating may impair endothelial function both acutely and chronically. High‐fat meals acutely impair endothelial function, a key early event in atherogenesis (Fewkes et al. 2021; Vogel et al. 1997; Bae et al. 2001; Fard et al. 2000; Giannattasio et al. 2004), which is likely to be mediated by changes in circulating lipoproteins, oxidative stress, inflammation and pro‐thrombotic factors (Higgins and Adeli 2017; Borén et al. 2014; Ansar et al. 2011). The chemical changes that occur following repeated heating may further impair postprandial endothelial function. However, to date, there are only two studies in humans that have investigated this with mixed results (Williams et al. 2001; Rueda‐Clausen et al. 2007).

This study aimed to assess the acute difference in vascular function following a meal rich in heated oil compared to a meal with unheated oil in healthy men. The primary outcome was endothelial function measured using flow‐mediated dilation (FMD); secondary outcomes included arterial stiffness and postprandial lipids. It was hypothesized that repeatedly heating oil would result in a greater impairment in endothelial function compared with a corresponding unheated oil.

## 2 Methods

The study was conducted between January and September 2007 at the Clinical Pharmacology Department, St. Thomas' Hospital, London, with ethical approval obtained from King's College London Research Ethics Committee (CREC/06/07‐45). The study was conducted in accordance with the ethical standards of the Declaration of Helsinki. All participants provided written informed consent. The trial was not registered in a clinical trial database, as it was conducted prior to this being standard practice.

### 2.1 Study Design and Participants

Twenty‐one men aged 18–40 years were recruited from the staff and students at King's College London. Participants were healthy and exclusion criteria included smoking, BMI > 35 kg/m^2^, fasting cholesterol > 7.8 mmol/L, triglycerides > 3 mmol/L, cardiovascular disease, diabetes, abnormal liver function, a history of myocardial infarction, angina, venous thrombosis, stroke, diabetes (or fasting plasma glucose > 7 mmol/L) or cancer, recent use of anti‐hypertensive medication, recent use of oral hypolipidaemic therapy, currently taking medication for regulating haemostasis such as heparin, a gastrointestinal disorder which may alter absorption or motility, having been exposed to any investigational agent within 30 days of the study and self‐reported high alcohol intake > 28 units/week. Habitual nutrient intake was assessed from a 3‐day food intake diary, and nutrients were estimated using the Microdiet program (Downlee Systems Limited). Dietary intakes were close to the recommended dietary guidelines for the UK (Henderson et al. 2003).

A randomized (computer‐randomization (EPISTAT, Uppsala, Sweden)), single‐blind, crossover study was conducted to compare postprandial responses to meals containing 50 g of heated versus unheated oil. Each participant consumed the two test meals on separate study days, with a minimum 1‐week washout. On the day preceding each postprandial test, participants were advised to avoid consuming foods high in fat and were provided with a standardized low‐fat dinner (containing < 10 g fat) to consume as their evening meal. Participants were asked to refrain from strenuous exercise, including cycling and sporting activities, and from the use of alcohol and caffeine on the day before and the day of the test meal. Participants fasted overnight from 2200 h and the following morning, between 0800 h and 1000 h, fasting venous blood samples were obtained. Anthropometric measurements (height, weight, waist/hip circumference), body composition via bioelectrical impedance (Tanita) and triplicate blood pressure readings (Omron 705 CP) were taken. Following a 15‐min rest, vascular function measurements [FMD of the brachial artery and pulse wave analysis (PWA)] were conducted (methods detailed below). The test meal was consumed within 15 min, and further venous blood samples were obtained 3 and 4 h later. Assessment of FMD and PWA was repeated between the 3 and 4 h blood samples and following the administration of 25 μg glycerol trinitrate (GTN) sublingually (to assess endothelium‐independent vasodilation). During the postprandial period, participants refrained from the consumption of any food or drink except water, which they were asked to sip at regular intervals throughout. The study was powered to detect a 2.1% change in FMD with 80% power at *p* < 0.05 (based on a 2% within‐subject SD) (Berry et al. 2008), resulting in a target sample size of 16 participants. Twenty‐one participants were recruited to allow for dropouts.

### 2.2 Test Meals

Test meals consisted of muffins containing 50 g of fat in the form of either unheated or repeatedly heated oil: 44% high oleic sunflower oil, 26% sunflower oil, 30% palm olein (from PURA Foods Ltd). The test fat was selected to represent an oil blend commonly used by the UK catering market, which provides an optimum fatty acid composition for oil stability, health and consumer‐acceptable taste. The fatty acid composition of the fat was: 16:0; 14%, 18:0; 4%, 18:1; 56% and 18:2; 24%. The heated oil underwent commercial deep‐frying conditions: 10 consecutive days with the temperature held at 180°C for the duration of the day and with 5 batches of chips fried daily (50 portions total). This is the heating protocol used by a leading culinary oil company (ADM Ltd) to assess the stability of their oils for commercial catering applications, including frying. The heated and unheated oil had 23.8% and 7.2% polar compounds, 4.41 mm/kg and 1.01 mm/kg peroxide value, and 0.59% and 0.04% free fatty acids, respectively. Total polar compounds and secondary oxidation products are used as indicators of oil degradation and safety (with many European countries limiting total polar compounds to 25% in frying oils (Sebastian et al. 2014)).

Each test meal was supplemented with vanilla milkshake (220 mL, 56 kcal, 1 g fat) and low‐fat custard (102 kcal, 1 g fat) for blinding and palatability purposes. The nutrient profile of the meal (muffin, milkshake and custard) was 52 g fat, 15 g protein, 81 g carbohydrate and 849 kcal.

#### 2.2.1 Blood Collection and Processing

Venous blood samples were collected (0, 3 and 4 h) via vacutainer technique and processed within 15 min of blood collection. Blood for lipid (TAG and NEFA), vitamin E and glucose analysis was collected into 6 mL EDTA and 4 mL fluoride oxalate‐containing vacutainers respectively and plasma was separated by centrifugation at 1500 g, 15 min at 4°C. All samples were stored at −80°C until analysis.

### 2.3 Outcomes

#### 2.3.1 Vascular Function Assessment

All vascular measurements were performed with the subject supine in a quiet, darkened, and temperature‐controlled (23°C) room. For full details of analysis, see Berry, Tucker, Banerji, et al. In brief, PWA was performed using the SphygmoCor apparatus (AtCor Medical, version 7.01) at the radial artery to assess arterial stiffness via augmentation index. FMD was measured in the brachial artery using high‐resolution ultrasound (7 MHz linear transducer, Siemens Acuson 128XP) following International Brachial Artery Reactivity Task Force Guidelines (Corretti et al. 2002). After baseline diameter measurement, hyperaemia was induced by 5‐min cuff occlusion. Arterial diameter was measured at 1‐ and 5‐min post‐deflation. Endothelium‐independent dilation was assessed using 25 μg sublingual glyceryl trinitrate (GTN) with measurement at 3 min, as a methodological control. GTN acts directly on the vascular smooth muscle to cause vasodilation and therefore induces vasodilation independently of a healthy functioning endothelium. All measurements were performed by blinded operators with triplicate readings.

#### 2.3.2 Analytical Methods

Vitamin E was quantified using the method of Thurnham et al. (1988) by high‐performance liquid chromatography (HPLC). Plasma (0.25 mL) was mixed with 0.25 mL internal standard solution, extracted with 1.0 mL heptane, and centrifuged at 2500 rpm for 10 min at 20°C. The organic phase was collected, dried under nitrogen at room temperature, reconstituted in chloroform, and transferred to amber HPLC vials for analysis.

Plasma glucose, TAG, and non‐esterified fatty acids (NEFA) were analyzed using enzymatic colourimetric assays on an ILAB‐650 clinical chemistry analyzer (Instrumentation Laboratories) as described elsewhere (Sanders et al. 2011).

### 2.4 Statistical Analysis

Data were assessed for normality by examining residuals from the ANOVA models using both the Shapiro–Wilk test and visual inspection of Q–Q plots. Postprandial data were analyzed using repeated‐measures ANOVA, with diet and time as within‐subject factors and meal order as a between‐subjects factor. Baseline brachial artery diameter was included as a covariate in the model for FMD analysis. When significant main effects were identified, post hoc comparisons were conducted using Bonferroni‐adjusted paired *t*‐tests. GTN‐induced vasodilation between meals was compared using a paired *t*‐test. To examine associations between postprandial TAG responses and vascular function, Spearman's rank correlation coefficients were calculated between changes in TAG and %FMD. Statistical analyses were performed using SPSS for Windows (version 31, IBM, Armonk, NY, USA). A two‐tailed *p* < 0.05 was considered statistically significant.

## 3 Results and Discussion

A total of 19 participants completed the study out of 21 who were randomized to treatment (1 withdrew due to time constraints and 1 was unable to consume the test meal). Table 1 shows participant characteristics of the study.

**TABLE 1:** Participant characteristics^a^.

Age (y)	22.4 ± 3.69
Height (cm)	179 ± 7.35
Weight (kg)	73.4 ± 11.1
BMI (kg/m^2^)	22.6 ± 2.26
Waist circumference (cm)	82.1 ± 6.85
Hip circumference (cm)	99.9 ± 4.19
Waist: hip ratio	0.82 ± 0.05
Serum total cholesterol (mmol/L)	4.43 ± 0.61
Serum TAG (mmol/L)	1.08 ± 0.39
HDL cholesterol (mmol/L)	1.36 ± 0.23
LDL cholesterol (mmol/L)	2.53 ± 0.60
Fasting plasma glucose (mmol/L)	5.06 ± 0.33
Systolic blood pressure (mmHg)	125 ± 10.1
Diastolic blood pressure (mmHg)	70.1 ± 7.62
Percentage body fat (%)	13.5 ± 3.72
Dietary intake	
Energy (MJ/d)	6.6
Protein (% energy)	16.4
Carbohydrate (% energy)	42.8
Total fat (% energy)	39.7

^a^ Vales are means ± SD, *n* = 19.

### 3.1 Vascular Function and Arterial Stiffness

FMD remained unchanged after both meals and was not different between meals (Table 2). This unexpected finding is discussed below after the lipid results to provide context. Brachial artery diameters at baseline and following reactive hyperaemia did not differ between meals or time points. However, a significant diet × time × order interaction was observed for post‐hyperaemia diameter (*p* = 0.012), indicating that the time course of vascular responses differed between diets depending on the order in which they were consumed. Endothelium‐independent vasodilation, in response to GTN, did not differ between meals. There was a significant time effect for both indices of arterial stiffness (PAIx and CAIx). Post‐hoc testing showed that PAIx reduced at 3 h postprandially after the heated oils meal, but changes from baseline were not different between meals. These reductions in arterial tone reflect normal physiological responses to meal ingestion and are similar to those observed in our previous research using a similar test meal model (Berry et al. 2008). Collectively, these data suggest that the consumption of repeatedly heated oil did not acutely impair endothelial function in healthy men.

**TABLE 2:** Postprandial vascular function, plasma lipids, glucose and vitamin E before and after meals containing heated or unheated oil^a^.

	Heated					Unheated					*p* ^b^		
	0 h	3 h	4 h	*Δ* 3–0 h	*Δ* 4–0 h	0 h	3 h	4 h	*Δ* 3–0 h	*Δ* 4–0 h	D	T	D × T
Baseline diameter (mm)	3.61 (3.34, 3.88)	3.59 (3.33, 3.85)		0.00 (−0.07, 0.77)		3.62 (3.35, 3.88)	3.62 (3.36, 3.90)		−0.02 (−0.04, 0.08)				
Post‐reactive diameter (mm)	3.88 (3.62, 4.12)	3.86 (3.59, 4.12)		−0.02 (−0.10, 0.064)		3.986 (3.60, 4.12)	3.87 (3.60, 4.12)		0.01 (−0.06, 0.08)				
FMD (%)	8.29 (6.41, 10.18)	7.67 (6.15, 9.19)		−0.62 (−2.13, 0.87)		7.09 (5.05, 9.14)	6.78 (4.88, 8.67)		−0.31 (−1.33, 0.71)				
GTN (%)		11.97 (10.36, 13.59)					10.91 (9.22, 12.61)						
PAIx (%)	43.26 (37.53, 48.99)	37.84 (32.09, 43.59)*		−5.42 (−9.45, −1.39)		44.16 (37.60, 50.72)	40.53 (34.25, 46.81)		−3.63 (−8.04, − 0.77)			0.008	
CAIx (%)	−9.58 (−14.38, −4.77)	−12.95 (−17.68, −8.21)		−3.37 (−6.53, −0.21)		−5.74 (−13.76, 2.29)	−10.68 (−15.80, −5.57)		−4.95 (−11.94, 2.05)			0.011	
TAG (mmol/L)	1.13 (0.90, 1.37)	1.58 (1.27, 1.90)^†^*	1.78 (1.45, 2.11)^†^*	0.45 (0.18, 0.72)^‡^	0.65 (0.39, 0.90)^‡^	1.02 (0.82, 1.22)	2.00 (1.64, 2.35)*	2.25 (1.84, 2.65)*	0.98 (0.70, 1.26)^‡^	1.23 (0.88, 1.58)^‡^		< 0.001	0.001
NEFA (mmol/L)	0.44 (0.36, 0.51)	0.28 (0.22, 0.35)*	0.44 (0.38, 0.50)	−0.15 (−0.25, −0.05)	0.01 (−0.06, 0.08)	0.40 (0.31, 0.50)	0.28 (0.22, 0.33)*	0.41 (0.36, 0.47)	−0.13 (−0.24, 0.01)	0.01 (−0.11, 0.13)		< 0.001	
Glucose (mmol/L)	4.84 (4.50, 5.19)	4.19 (3.81, 4.57)*	4.64 (4.3, 4.96)*	−0.65 (−0.90, −0.41)	−0.21 (−0.44, 0.03)	4.89 (4.52, 5.26)	4.06 (3.63, 4.49)*	4.73 (4.37, 5.09)	−0.83 (−1.30, −0.36)	−0.16 (−0.50, 0.19)		< 0.001	
Vitamin E (μmol/L)	20.9 (14.6, 27.1)	24.2 (16.7, 31.7)	25.9 (19.3, 32.5)	3.3 (0.6, 6.1)	5.03 (0.73, −9.33)	24. (18.5, 30.6)	22.2 (17.2, 27.3)	26.0 (20.2, 31.7)	−2.4 (−5.6, 0.9)	1.4 (−3.8, 6.5)			

^a^ Values are means (95% CI), *n* = 19. *p < 0.05, within‐meal change versus baseline (Bonferroni corrected), ^†^ *p* < 0.05 versus unheated meal at the same time point (Bonferroni corrected), ^‡^ *p* < 0.05 change from baseline versus unheated meal (Bonferroni corrected).

^b^ Calculated using repeated‐measures ANOVA. D, Diet effect; T, time effect; D × T, diet × time interaction.

### 3.2 Postprandial Plasma Lipids and Glucose

**FIGURE 1:**
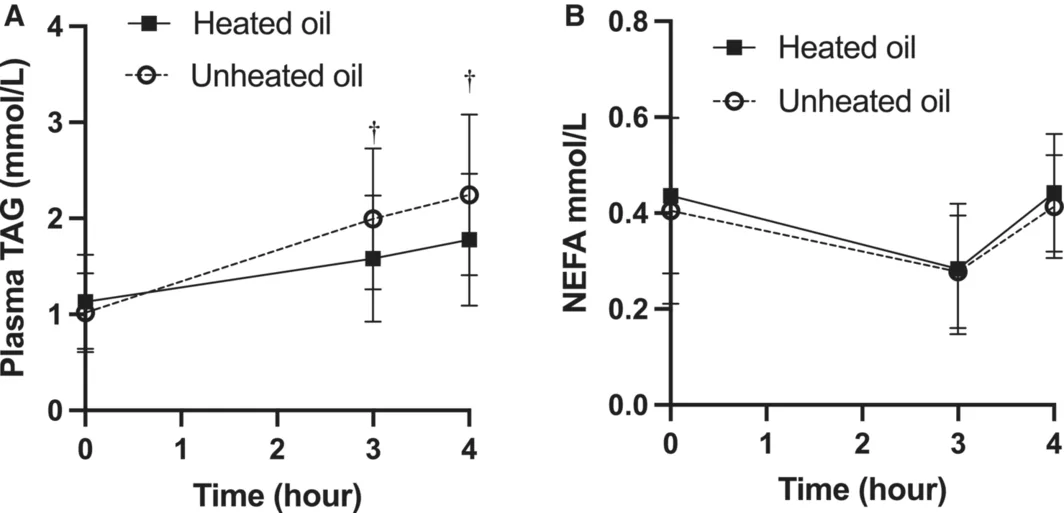
Plasma triglyceride (TAG) (A) and non‐esterified fatty acid (NEFA) (B) concentrations in healthy men after consumption of a meal containing heated or unheated oil. Deviations from fasting were analyzed by repeated measures ANOVA. Values are mean (SD). For TAG, Time effect, *p* < 0.001; Diet × Time effect, *p* < 0.001. For NEFA, Time effect, *p* < 0.001. ^†^Different from unheated at the corresponding time point, *p* < 0.001.

Plasma TAG concentrations (Figure 1A and Table 2) at 3 and 4 h were lower after the heated oil meal (1.6 and 1.8 mmol/L respectively) compared with the unheated oil meal (2.0 and 2.3 mmol/L respectively) (meal × time interaction, *p* = 0.001). There were no differences in the postprandial change in NEFA (Figure 1B) or glucose (Table 2) between meals. Postprandial changes were of a similar magnitude to those observed in our previous studies utilizing a similar test meal composition (Hall et al. 2025; Berry, Valdes, et al. 2020).

The lower TAG response after the heated oil meal may be due to thermal degradation of the oil, as evidenced by the difference in polar compounds between the heated and unheated oil (23.8% vs. 7.2% respectively), which may impact intestinal absorption and metabolism. Indeed, the heating process forms oxidized lipids, polymers, and free fatty acids, which are less readily hydrolysed and absorbed compared to native fatty acid structures in unheated oils (Thilakarathna et al. 2019; Ye et al. 2019). In contrast, unheated oils maintain their native TAG structures, which are generally more readily hydrolysed and absorbed, leading to a more rapid rise in circulating TAG in animal studies (Aja et al. 2022; Bao et al. 2025; Ruan et al. 2020; Zhang et al. 2023). However, the clinical relevance of the size of difference in postprandial increases in TAG between meals (mean difference of 0.6 mmol/L) is uncertain.

These findings are consistent with Williams et al. (2001), who also reported lower TAG after heated compared with unheated oil at 4 h postprandially. In contrast, Rueda‐Clausen et al. (2007) observed similar postprandial TAG increases across high‐fat meals regardless of oil heating or frying level. This lack of difference observed by Rueda‐Clausen may be due to the relatively low polar compound value (9% max) in the heated oils compared to the present study (24%), as well as the oil type, heating conditions, or meal matrix. Interestingly, rodent studies have generally shown that repeatedly heated oils lead to elevated plasma TAG (Seema et al. 2023; Kwape et al. 2024; Chacko and Thankappan 2020; Ochiai 2020), highlighting the importance of interpreting the health effects of oils on human health from animal studies with caution.

Plasma vitamin E concentrations did not differ significantly between meals or time points, with levels remaining stable across the postprandial period (Table 2). This contradicts available animal evidence; rats and pigs fed repeatedly heated or peroxidised oils show reduced serum vitamin E and impaired antioxidant status (Silva‐Guillen et al. 2019, 2020; Umoh et al. 2024) which could be attributed to a lower vitamin E concentration in heated oils versus their unheated counterparts (Kuppithayanant et al. 2014). Unfortunately, the vitamin E concentration was not measured in the heated and unheated oil before consumption in the current study, so it is not possible to explain the lack of difference observed. However, the vitamin E concentration of a similar oil, under similar conditions (whereby the polar compounds also changed from ~7% to ~25% and the peroxide value from 0.8 to ~5 mg/kg upon repeated heating) changed from ~45 to ~9 mg/kg (analysis undertaken by PURA Foods Ltd).

It was hypothesized that both test meals would impair endothelial function, with a greater impairment predicted after the heated oil due to the generation of oxidation products, polar compounds and peroxides. Whilst it is plausible that a potentially greater postprandial impairment of FMD following the heated oil may have been offset by the lower postprandial TAG response compared to the unheated oil, the lack of change in postprandial FMD following both oils was unexpected. Early studies by Vogel et al. (1997), demonstrated a large reduction in FMD following high‐fat meals, which was likely mediated by the increase in circulating TAG and oxidative mechanisms. Subsequent randomized controlled trials have reported inconsistent results regarding the postprandial impairment of FMD (Berry, Tucker, Banerji, et al. 2008; Raitakari et al. 2000; Lacroix et al. 2013; Rathnayake et al. 2018; Markey et al. 2021). In the current study, Spearman's correlation revealed that there was no relationship between FMD% and TAG (heated: $r_{s}=-0.24$, *p* = 0.32; unheated: $r_{s}=-0.12$, *p* = 0.63). Additionally, although Williams et al. (2001) observed a greater impairment of FMD following a heated versus unheated oil (Williams et al. 2001), Rueda‐Clausen et al. (2007) found that heated and unheated oils resulted in a similar level of reduction in postprandial FMD. Both studies included healthy men, however the greater age of the participants in the Williams et al. study (mean; 38 years) versus the Rueda‐Clausen et al. study (mean; 21 years), as well as differences in oil type, heating method, polar compound content, and analytic covariates (e.g., baseline diameter) may explain the discrepancies. The inclusion of young (mean age 22.4 years) healthy men may provide one possible explanation for the lack of change observed in the current study.

These results warrant further investigation, given the widespread use of heated vegetable oils (such as rapeseed/canola, cottonseed, palm, soybean, corn, or sunflower oil) in frying and high‐heat cooking in many western populations (Mazzocchi et al. 2019). Indeed, the animal and mechanistic evidence shows that repeatedly heating oils impairs endothelial function, and the human data suggest a risk of endothelial damage with chronic exposure. Additionally, given the unsubstantiated narrative that seed oils may be “pro‐inflammatory” due to their high linoleic acid content, it would be timely to investigate the effects of repeated heating of seed oils versus oils higher in saturated fat (such as beef tallow and palm oil).

### 3.3 Limitations

The timing of postprandial measurements (0, 3, and 4 h) limited the resolution of earlier or later lipid dynamics, which may peak at 3–6 h in some individuals. Additionally, the study population consisted of young, healthy men, which restricts the applicability of the findings. Given the rising prevalence of metabolic disorders, this limits the generalisability of the results to broader and more clinically relevant populations. Future studies should include metabolically unhealthy individuals of both sexes, given the greater adverse metabolic impact of fat‐containing meals in these populations. Whilst the levels of polar compounds (gold standard commercial measure of oil oxidation/quality) and peroxides were measured, we did not measure other chemicals that repeat heating may affect (e.g., aldehydes, ketones, dimers and polycyclic aromatic hydrocarbons and acrylamide). This partially limits the comparison of the final heated oil tested in this study to that used in commercial deep fryers. Despite the time lapse between conducting and publishing the trial, the research question remains relevant, and the dataset provides valuable insight into the oils most commercially used previously and currently for deep frying applications.

## 4 Conclusion

In summary, despite a difference in the postprandial rise in plasma TAG between heated and unheated oil, there was no difference in vascular function and arterial stiffness. These findings suggest that repeated heating of oil modifies postprandial lipid handling but does not acutely impair vascular function. Given the inconsistency of results across prior human and animal studies, further controlled trials with larger sample sizes, detailed characterization of oil chemical modifications, and extended postprandial monitoring are required to clarify the metabolic and vascular implications of consuming repeatedly heated oils.

## Author Contributions

Sarah Berry and Wendy Hall conceived and designed the study. Wendy Hall, Sarah Berry, and Rosiered Brownson‐Smith wrote the first draft of the manuscript. Sarah Berry, Wendy Hall, Sarah Fissler, Jo Bruce and Esmat Abbas carried out the research, and Rosiered Brownson‐Smith, Sarah Fissler and Esmat Abbas analyzed the data. All authors contributed to and approved the final draft of the manuscript.

## Conflicts of Interest

Sarah Berry and Wendy Hall have received funding from the Malaysian Palm Oil Board. Jo Bruce is employed by ADM (UK) Ltd.

## Data Availability

The data that support the findings of this study are available from the corresponding author upon reasonable request.
